# Joint Action of the Medical Society of the County of Erie and the Buffalo Academy of Medicine

**Published:** 1908-11

**Authors:** 


### Joint Action of the Medical Society of the County of Erie and the Buffalo Academy of Medicine.

*To the Honorable, the Board of Aldermen, of the city of Buffalo:*

We, the undersigned, being representatives of the Medical Society of the County of Erie, and the Buffalo Academy of Medicine, appointed for the purpose, do hereby petition your honorable body to take immediate steps for the establishment and maintenance of a city hospital for infectious and contagious diseases. The reasons for this request are as follows:

First.—During the year from July 1, 1907, to July 1, 1908, there occurred in the city of Buffalo, the following number of cases of contagious diseases: measles, 2,388; scarlet fever, 1,324; diphtheria, 440; and the following number of deaths from measles, 46; scarlet fever, 59; diphtheria, 65; distributed through the different months as per the attached table. There were per 10,000 inhabitants, measles, 60; scarlet fever, 35; diphtheria, 11.

Second.—The present hospital facilities in Buffalo for receiving such cases are utterly, woefully and totally inadequate—the Sister's Hospital being able to accommodate but 10, the Children's Hospital, 10 (their charter does not require them to take contagious diseases, and their beds are only for small children), Erie County Hospital, 8. These, we believe, are the only institutions which will receive these cases and none of these are arranged on the pavilion system, so that the care of different kinds of contagious diseases at the same time is dangerous. These accommodations give a total capacity of 30, or one bed for every 13,330 inhabitants.

Third.—Glasgow has hospital beds for contagious diseases 9.79 for every 10,000 inhabitants; London, Eng., has 9; Boston has 3.85.

Fourth.—When so quarantined, social or business relations are either entirely suspended or greatly diminished. While such quarantine, if strictly enforced, is of the greatest value to the health of the city at large, and, therefore, should not in any way be interfered with, still it is broken unless enforced by a large number of police, to the detriment of public health, as in the present epidemic of scarlet fever. It is a hardship which will be to a great extent obviated by the establishment of such a hospital. For example, if a case of such infectious disease occur in a hotel or boarding house, such hotel or boarding house must be quarantined and placarded; if a domestic employee comes down with such disease, the house in which such employee lives must be quarantined, and the children of the family interfered with. Some families have the shop and home in the same building, in such case, the business would be entirely stopped and the source of income ceases. If such case occurs in the family of a woman who goes out to work, her means of livelihood is stopped, and many times a physician is not called for fear of quarantine, resulting in the spreading of the disease. If such case occur in a tenement house, the spread of disease is enormous and the impossibility of proper quarantine is evident.

Fifth.—The results of treatment of these cases in hospitals are much better than in private practice, both as to diminished death-rate and in fewer serious sequels.

Sixth.—As a result of removal of such cases to hospitals, sources of infection are removed from the people and a fewer number of cases occur in the community as proven by statistics of those places where such institutions exist—notably in Glasgow, Scotland; London, Eng.; Boston, Mass.; and New York city. The figures for these places, before and after the establishment of these hospitals, are at hand if desired.

With these facts before you, we believe it unnecessary to present any prolonged argument as to the necessity of taking steps for the immediate establishment of such a hospital.

Committee of the Medical Society of the County of Erie on necessity for a city hospital for infectious diseases:

John D. Bonnar, DeLancey Rochester,
Herman E. Hayd, Lawrence G. Hanley.

Commission on contagious diseases and municipal contagious hospital, of the Buffalo Academy of Medicine:

Julius Ullman, Allen A. Jones,
DeWitt H. Sherman, Grover W. Wende,
Robert F. Sheehan, Jr.

